# Remarks on the Review of a Paper in the Philosophical Transactions

**Published:** 1816-06

**Authors:** A. P. Wilson Philip

**Affiliations:** Worcester.


*For the London Medical and Physical Journal.*

*Remarks on the Review of a Paper in the Philosophical Transactions;* by A. P. Wilson Philip, M.D. of Worcester.

IN the London Medical and Physical Journal for last April, a gentleman who had reviewed a paper of mine, published in the Philosophical Transactions of last year, replies, in a letter addressed to the Editors, to certain charges brought against his account of it. It is not my intention to make any observations either on his account of my paper or his reply to those charges: of both the public will judge. I wish to inquire what motive has induced him to attempt, by misrepresentation, to substantiate a charge of cruelty against me. I do not allude to his frequently calling the reader's attention to the cruelty of such experiments as those related in my paper, without at all adverting to the means I employed ployed to lessen the sufferings they occasion, which all will allow to be uncandid;—I allude to more direct language. In one passage of the above letter he says, "Without repeating the disgusting cruelties of hot wires applied to the spinal marrow, of various incisions, ligatures, &c." Hot wires were, indeed, frequently applied to the spinal marrow in my experiments, but this gentleman conceals the circumstance that it was to the spinal marrow of the dead animal alone that they were applied. He also says, after stating that my experiments may lead to some important conclusions, "I sincerely hope, therefore, that Dr. P. will continue his experiments with less cruelty." After such an observation, it is incumbent on this gentleman to point out how my experiments could have been made with less cruelty.

My anxiety to lessen the sufferings of animals in such experiments induced me, on a former occasion, publicly to propose the following rules for this purpose, which I beg leave here to repeat:—To destroy the sensibility of the animal previous to the experiment, when this can be done without influencing the result. When more than one animal are equally fit for the experiment, to choose that which will suffer least by it. To destroy the animal as soon as the purpose in view is answered; and to take such precautions as shall render as few repetitions of the experiment as possible necessary.—To these rules I have, in every part of my investigation, strictly adhered. If the author of the above letter can point out any addition to them, tending farther to lessen the sufferings necessarily attending such investigations, I shall be happy to express my thanks for the communication of it. It cannot surely be maintained that no such experiments should be made, in which the most important interests of human nature may be concerned.

All who assisted me in my experiments will declare, that I never failed to expatiate on the cruelty of experiments on living animals, and on the necessity of weighing well the objects in view before such experiments are undertaken; and that many points which both they and I wished to ascertain were left undetermined, wholly because they were not considered of sufficient importance to sanction the sufferings which the inquiry would occasion.

The observations with which you preface the letter above referred to, have induced me to address these observations to you, rather than to the editors of any other journal; and to request that you will do me the justice to give them a place in your Journal for next month.

*Worcester; May* 11, 1816.

